# Risk factors for level V lymph node metastases in solitary papillary thyroid carcinoma with clinically lateral lymph node metastases

**DOI:** 10.1002/cam4.792

**Published:** 2016-07-01

**Authors:** Jing Yang, Yanping Gong, Shuping Yan, Jingqiang Zhu, Zhihui Li, Rixiang Gong

**Affiliations:** ^1^ Department of Thyroid and Breast SurgeryWest China HospitalSichuan UniversityNo. 37 Guo Xue XiangChengduSichuan610041China

**Keywords:** Lymph nodes, neck dissection, papillary, risk factors, thyroid cancer

## Abstract

The extent of lateral neck dissection (LND) in surgical resection of papillary thyroid carcinoma (PTC) with clinically lateral LNM (LLNM) remains controversial. We aimed to explore the frequency of and risk factors for level V LNM in patients with solitary PTC and clinically LLNM. To analyze the frequency and risk factors for level V LNM, we retrospectively reviewed 220 solitary PTC patients who underwent total thyroidectomy, bilateral central neck dissection, and therapeutic LND. LLNM were present in 82.3% patients, and levels II–V LNM were present in 45.9%, 62.7%, 55.5%, and 12.3% patients, respectively. Ipsilateral level V LNM was significantly associated with tumor size >10 mm, extrathyroidal extension, ipsilateral central LNM ratio ≥50%, and contralateral central LNM (CLNM), bilateral CLNM, and simultaneous levels II–IV LNM. Contralateral CLNM was an independent risk factor for level V LNM. In patients with solitary PTC and clinically LLNM, level V LNM was relatively uncommon. Therefore, routine level V lymphadenectomy may be unnecessary in these patients unless level V LNM is suspected on preoperative examination or associated risk factors, especially contralateral CLNM, are present.

## Introduction

Cervical lymph node metastases (LNM) in papillary thyroid carcinoma (PTC), the most common histological type of thyroid cancer with an increasing worldwide incidence 1, are frequent and occur in approximately 30–80% patients 2, 3. Cervical LNM in PTC have been identified as an independent risk factor for regional recurrence 4, 5, 6, 7, and emerging evidences from large population‐based studies have indicated decreased disease‐free survival rate and increased mortality associated with regional LNM 7, 8, 9, 10. There is universal agreement that therapeutic lateral neck dissection (LND) should be undertaken in patients with PTC and clinically lateral LNM (LLNM) on the basis of palpation or imaging examination 11, 12. However, determining the appropriate extent of LND remains controversial. Radical operations, such as those with increased extent of LND, may lead to clinically important postoperative morbidities (shoulder dysfunction, neck numbness, and neuropathic pain) because of injury to the spinal accessory nerve or the cervical plexus despite gross preservation of these nerves 13, 14, 15. Therefore, an oncologically effective therapeutic LND is critical to postoperative outcome.

In general, the extent of therapeutic LND includes levels II–V. However, it is debatable whether routine level V lymphadenectomy is necessary in patients with PTC with clinically LLNM 16, 17, 18, 19, 20, 21, 22. In addition, there have been few studies to explore the risk factors for level V LNM in solitary PTC with clinically LLNM. To determine a correlation that could define the rational extent of therapeutic LND in PTC, we aimed to explore the frequency of and the risk factors for level V LNM in solitary PTC patients with clinically LLNM.

## Patients and Methods

### Study population

We retrospectively reviewed the medical records of consecutive patients with histologically proven solitary (without pathological evidence of multifocality) PTC who underwent simultaneous total thyroidectomy (TT), bilateral central neck dissection (CND), and LND (at least from levels II to V) at the Department of Thyroid and Breast Surgery, West China Hospital of Sichuan University, between January 2011 and December 2014. All patients underwent preoperative physical examination, high‐quality thyroid ultrasonography (US), and US‐guided fine‐needle aspiration biopsy (USgFNAB) of the primary tumor. At our institution, preoperative US is routinely performed to access cervical lymphadenopathy, with therapeutic LND performed mostly on the basis of the US findings; therefore, our study included solitary PTC patients with clinically LLNM suspected on US according to at least one of the following metastatic criteria: round shape (long/short ratio <2), microcalcification, cystic change, hyperechogenicity, and heterogeneous inner structure 23. The final diagnosis of primary tumors and cervical LNM was based on pathological examination of surgical specimens. Patients were excluded from the study if they had thyroid carcinoma with a diffuse sclerosis variant, mixed histology, cancer localized to the isthmus, reoperation, or undivided lymph node specimens. Consequently, 220 patients were enrolled in the study. Of these, 25 and 195 patients had undergone bilateral and unilateral LND, respectively. This study was approved by the institutional review board of the West China Hospital of Sichuan University.

### Tumor and lymph node classification

In our study, solitary PTC was defined as PTC without pathological evidence of multifocality within the thyroid. The largest diameter and location of the primary tumor within the thyroid were determined from pathology reports. The location of the tumor was classified according to which third (superior, middle, or inferior) of the affected thyroid lobe was involved. If a tumor extended into an adjacent lobe, it was categorized according to all sections involved 24.

**Figure 1:**
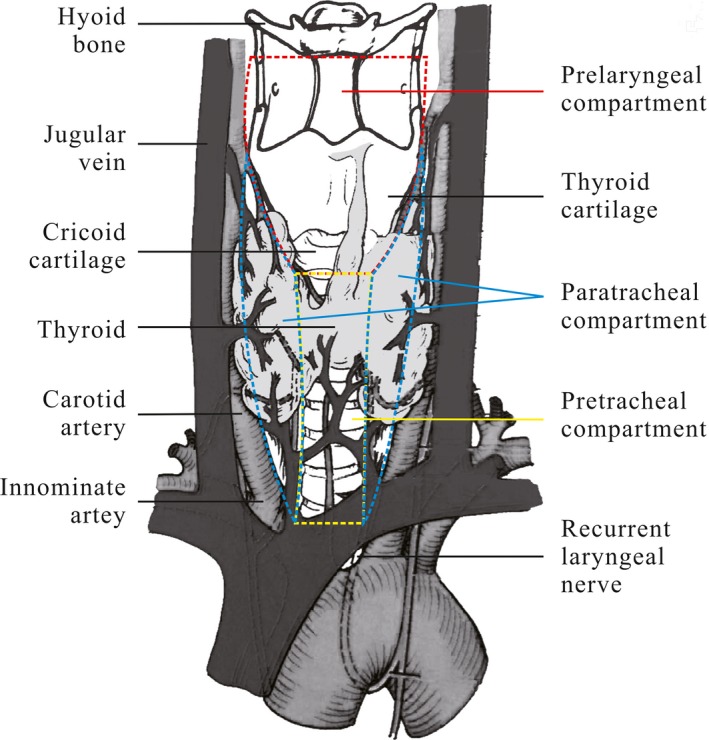
Subgroups of the central compartment (Prelaryngeal lymph node demarcated superiorly by the inferior border of the hyoid bone and inferiorly by the inferior border of the cricoid cartilage; Ipsilateral/contralateral paratracheal lymph node demarcated laterally by the carotid artery and medially by the trachea; Pretracheal lymph node demarcated superiorly by the inferior border of the cricoid cartilage and inferiorly by the innominate artery) 210x297mm (300 x 300 DPI).

The entire thyroid gland was excised first, followed by bilateral CND and LND. The maximum extent of CND was the hyoid bone superiorly, the innominate vein inferiorly, and the carotid sheaths laterally. Central lymph node (CLN) specimens were classified as prelaryngeal, pretracheal, ipsilateral paratracheal, or contralateral paratracheal (Figure 1). Next, the prelaryngeal, pretracheal, and ipsilateral paratracheal lymph nodes were defined as ipsilateral CLN and the contralateral paratracheal lymph node as contralateral CLN, according to the definition of laterality proposed by Keum et al. 22. The surgeon separately removed the nodes in each of these categories. The LND was carried out in the usual fashion from at least level II to V, sparing the internal jugular vein, spinal accessory nerve, and sternocleidomastoid muscle. The surgeon also separated the LND specimens according to neck levels. All thyroid and LND specimens were sent to the department of pathology for paraffin fixation and histological analysis. All neck dissection specimens were recorded according to neck regions, but only the ipsilateral lateral specimens that had a bilateral LND were analyzed in this study.

Information on the following risk factors was obtained: sex, age, size, and location of the primary tumor, capsular invasion, extrathyroidal extension, presence of coexistent lymphocytic thyroiditis, and the extent of LNM.

### Statistical analysis

In univariate analysis, categorical variables were analyzed using Pearson's chi‐square test and continuous variables were analyzed using the Student's *t*‐test or the Wilcoxon rank‐sum test. Binary logistic regression analysis was used for the multivariate analysis of categorical variables, with *P *<* *0.100 on univariate analysis; *P *<* *0.05 was considered statistically significant. Statistical analysis was performed using STATA version 12.0 (Stata Corporation, College Station, TX, USA).

## Results

Of the eligible patients with solitary PTC (*n *=* *220), 51 (23.2%) were male and 169 (76.8%) were female. The median age was 41.0 ± 13.5 years (range: 13–79 years) and the mean size of the primary thyroid tumor was 21.4 ± 13.3 mm (range: 3–89 mm). A summary of patient and tumor characteristics is shown in Table 1. LNM was histologically confirmed to involve the central compartment in 177 patients (80.5%) and the lateral compartment in 181 patients (82.3%). Twenty‐two patients (10%) had skip metastases (metastases to the lateral cervical compartment without metastasis to the central cervical compartment). Of the 177 patients with central LNM (CLNM), 172 patients (78.2%) had LNM in the central compartment ipsilateral to the primary tumor, 87 (39.5%) had LNM in the contralateral central compartment, and 82 (39.1%) had LNM in the bilateral central compartments. Of the 181 patients with LLNM, 44 patients (20.0%) had single‐level metastases, and 137 patients (62.3%) had multiple‐level metastases. Level III metastases were most common (138/220; 62.7%), followed by level IV (122/220; 55.5%), level II (101/220; 45.9%), and level V (27/220; 12.3%) metastases (Table 1). The mean ± SD (Standard Deviation) number of excised and metastatic lymph nodes in the central compartment was 10.19 ± 5.71 (range: 0–32) and 4.18 ± 3.90 (range: 0–20), respectively. The median total metastatic lymph node ratio was 0.41 ± 0.31 (range: 0.00–1.00) in the central compartment. In the lateral compartment, the mean ± SD number of excised and metastatic lymph nodes, respectively, was 24.95 ± 11.31 (range: 5–63) and 4.07 ± 3.79 (range: 0–23). The median total metastatic lymph node ratio was 0.18 ± 0.16 (range: 0.00–0.86) in the lateral compartment. The mean ± SD number of excised lymph nodes, metastatic lymph nodes, and median metastatic lymph node ratio in each compartment are shown in Table 2. The mean number of excised and metastatic lymph nodes and the median metastatic lymph node ratio were lower in contralateral compartment than those in ipsilateral compartment in the central compartment (*P *<* *0.001, Table 2). The mean number of excised and metastatic lymph nodes and the median metastatic lymph node ratio were lower in level V than those in level II, III, and IV in the lateral compartment (*P *<* *0.001, Table 2).

**Table 1:** Demographics and clinical characteristics of 220 solitary papillary thyroid carcinoma patients

Characteristics	Values (%)
No. of patients	220
Gender	
Male	51 (23.2)
Female	169 (76.8)
Age (years)	
Mean ± SD	41.0 ± 13.5
≥45	82 (37.3)
<45	138 (62.7)
Size (mm)	
Mean ± SD	21.4 ± 13.3
>10	170 (77.3)
≤10	50 (22.7)
Location of the primary tumor	
Superior lobe	111 (50.5)
Middle lobe	127 (57.7)
Inferior lobe	76 (34.5)
Capsular invasion	176 (80.0)
Extrathyroidal extension	99 (45.0)
Lymphocytic thyroiditis	48 (21.8)
Central lymph node metastases	177 (80.5)
Ipsilateral	172 (78.2)
Contralateral	87 (39.5)
Bilateral	82 (39.1)
Lateral lymph node metastases	181 (82.3)
Level II	101 (45.9)
Level III	138 (62.7)
Level IV	122 (55.5)
Level V	27 (12.3)
Single‐level	44 (20.0)
Multiple‐level	137 (62.3)

**Table 2:** The harvested lymph nodes in 220 solitary papillary thyroid carcinoma patients

Compartment	Excised number (Mean ± SD)	*P*‐value	Metastatic number (Mean ± SD)	*P*‐value	Metastatic ratio (Mean ± SD)	*P*‐value
Central lymph node						
Ipsilateral	6.34 ± 4.57	<0.001^b^	3.26 ± 3.33	<0.001^b^	0.50 ± 0.36	<0.001^a^
Contralateral	3.85 ± 3.01		0.92 ± 1.47		0.26 ± 0.37	
Lateral lymph node						
Level II	7.12 ± 4.31	<0.001^a^, ^c^	0.90 ± 1.29	<0.001^b^, ^c^	0.27 ± 0.29	<0.001^b^, ^c^
Level III	6.51 ± 3.99	<0.001^b^, ^d^	1.60 ± 1.79	<0.001^b^, ^d^	0.22 ± 0.29	<0.001^b^, ^d^
Level IV	7.36 ± 5.00	<0.001^b^, ^e^	1.38 ± 1.90	<0.001^b^, ^e^	0.22 ± 0.29	<0.001^b^, ^e^
Level V	3.95 ± 4.03	—	0.22 ± 0.94	—	0.04 ± 0.13	—

a The Student's *t*‐test was adopted.

b The Wilcoxon rank‐sum test was adopted.

c Level V versus Level II.

d Level V versus Level III.

e Level V versus Level IV.

Table 3 shows the association between level V LNM and several risk factors in the 220 solitary PTC patients who underwent LND for clinically LLNM. The mean age of the patients with level V LNM was similar to that of patients without level V LNM (Table 3). The mean size of the primary tumor in patients with level V LNM was larger than that in patients without level V LNM (Table 3). Univariate analysis showed that the presence of level V LNM was significantly associated with tumor size >10 mm, extrathyroidal extension, ipsilateral central LNM ratio ≥50%, and the presence of contralateral CLNM, bilateral CLNM, and simultaneous levels II + III + IV LNM (Table 3). Variables with *P *<* *0.100 were included in the multivariate logistic regression analysis. The multivariate analysis showed that only contralateral CLNM was an independent risk factor associated with level V LNM [odds ratio (OR): 14.001; 95% confidence interval (CI): 1.528–128.257; *P *=* *0.020; Table 4]. Gender, age, location of the primary tumor, capsular invasion, coexisting lymphocytic thyroiditis, ipsilateral CLNM, and level II, level III, level IV, simultaneous levels II + III and simultaneous levels III + IV LLNM were not found to be associated with level V LNM.

**Table 3:** Univariate analysis of risk factors related to level V lymph node metastases

Variables	Level V metastases		*P*‐value
	Present, *n* (%)	Absent, *n* (%)	
Total	27 (12.3)	193 (87.7)	
Gender			
Male	6 (22.2)	45 (23.3)	0.900
Female	21 (77.8)	148 (76.7)	
Age (years)			
Mean ± SD	41.9 ± 15.5	40.9 ± 13.2	0.708^a^
≥45	9 (33.3)	73 (37.8)	0.651
<45	18 (66.7)	120 (62.2)	
Size (mm)			
Mean ± SD	29.2 ± 20.7	20.3 ± 11.6	0.021^b^
>10	26 (96.3)	144 (74.6)	0.012
≤10	1 (3.7)	49 (25.4)	
Location of primary tumor			
Superior lobe	15 (55.6)	96 (49.7)	0.571
Middle lobe	19 (70.4)	108 (56.0)	0.156
Inferior lobe	11 (40.7)	65 (33.7)	0.470
Capsular invasion	24 (88.9)	152 (78.8)	0.218
Extrathyroidal extension	17 (63.0)	82 (42.5)	0.045
Lymphocytic thyroiditis	4 (14.8)	44 (22.8)	0.347
Central lymph node metastases			
Ipsilateral	24 (88.9)	149 (77.2)	0.165
Metastatic ratio ≥50%	21 (77.8)	107 (55.4)	0.028
Contralateral	17 (63.0)	70 (36.3)	0.008
Bilateral	15 (55.6)	67 (34.7)	0.036
Lateral lymph node metastases			
Level II	16 (59.3)	85 (44.0)	0.137
Level III	20 (74.1)	118 (61.1)	0.193
Level IV	18 (66.7)	104 (53.9)	0.211
Level II + III	13 (48.1)	68 (35.2)	0.193
Level III + IV	15 (55.6)	72 (37.3)	0.069
Level II + III + IV	11 (40.7)	41 (21.2)	0.026

a The Student's *t*‐test was adopted.

b The Wilcoxon rank‐sum test was adopted.

**Table 4:** Multivariate analysis of risk factors related to level V lymph node metastases

Variables	OR	95% CI	*P*‐value
Tumor size >10 mm	6.349	0.802–50.245	0.080
Extrathyroidal extension	1.418	0.575–3.495	0.448
Ipsilateral central lymph node metastatic ratio ≥50%	2.531	0.738–8.683	0.140
Contralateral central lymph node metastases	14.001	1.528–128.257	0.020
Bilateral central lymph node metastases	0.107	0.010–1.097	0.060
Level III + IV lymph node metastases	0.927	0.251–3.422	0.910
Level II + III + IV lymph node metastases	1.620	0.424–6.192	0.481

OR, odds ratio; 95% CI, 95 % confidence interval.

Additional subgroup analysis for 181 PTC patients with pathological LLNM by univariate analysis showed that level V LNM was significantly associated only with tumor size >10 mm (*P* = 0.030), and multivariate analysis showed that there was no independent risk factor associated with level V LNM.

## Discussion

Patients with PTC have excellent prognosis; however, the presence of LNM significantly increases the risk of locoregional recurrence, and some groups have demonstrated decreased disease‐free survival rate and increased mortality associated with regional LNM 4, 5, 6, 7, 8, 9, 10. Extensive LND has the potential to decrease regional recurrence and increase disease‐free survival, but may lead to clinically important postoperative morbidities 13, 14, 15. Therefore, determining a rational extent of therapeutic LND is vital. Whether level V should be included in therapeutic LND continues to be debated 16, 17, 18, 19, 20, 21, 22. A majority of previous studies had explored some predictors for level V LNM in PTC patients with clinically LLNM, but only a few studies have explored the risk factors for level V LNM in solitary PTC with clinically LLNM. Therefore, the frequency of and the risk factors for level V LNM in solitary PTC with clinically LLNM were analyzed to determine the rational extent of therapeutic LND.

Several imaging modalities, including US, computed tomography (CT), magnetic resonance imaging (MRI), and [^18^F]‐fluoro‐2‐deoxy‐D‐glucose‐positron emission tomography/CT (^18^FDG‐PET/CT) have been used to evaluate cervical LNM. However, US is more easily accessible and cheaper than other imaging modalities, and was determined to be the most sensitive method for assessing cervical LNM 25, 26. Therefore, US performed by an experienced ultrasonographer is considered, by most clinicians and by the American Thyroid Association, as the screening and surveillance imaging modality of choice for detecting LLNM 27. Although USgFNAC was showed to be the most specific and accurate imaging modality to detect cervical LNM 25, most studies report that USgFNAC has a relatively lower sensitivity than US, and the false‐negative rate of USgFNAC could be as high as 45–52% 28, 29. In addition, given the closer relationship of the node to the surrounding vascular structures and the body habitus of the patient (such as some small nodes, which are not easily punctured by fine needle), routine preoperative USgFNAC is not done to guide LND at our institution. In our study, the sensitivity of US was 82.3% (181/220) for predicting LLNM, and this is in the range of the sensitivities reported for US, with 63–97% specified in the meta‐analysis by de Bondt 25. The clinical evaluation of US that established the presence of LLNM in 17.7% (39/220) patients was inaccurate, which might have an inaccuracy that is lower than USgFNAC and other imaging modalities. Of course, USgFNAC and other imaging modalities would, to some extent, have helped prevent unnecessary LND in the 17.7% patients with a negative LLNM, but they might have contributed more to the omission of LND in patients with positive LLNM according to the reasons discussed earlier.

Our present study demonstrated that the mean number of excised and metastatic lymph nodes and the median metastatic lymph node ratio were lower in contralateral compartment than that in ipsilateral compartment in the central compartment, which were caused by that the ipsilateral CLM included prelaryngeal, pretracheal and ipsilateral paratracheal lymph nodes, and LNM generally follow a nearby principle. Secondly, the mean number of excised and metastatic lymph nodes and the median metastatic lymph node ratio were also lower in level V than that in other levels in the lateral compartment, which was consistent with the reported result by Lim et al. 16. Of course, those results might have been affected by the lower sampling lymph nodes on those areas. However, each compartment or level nodes were thoroughly swept by surgeons at our institution. Therefore, there was a smaller possibility to the lower sampling lymph nodes on those areas in our study.

Almost all previous studies have indicated that, in patients with PTC and clinical LNM, most LLNM were levels II, III, and IV. This is consistent with the findings of this study, which found that LLNM mainly occurred at levels II, III, and IV with frequencies of 45.9%, 62.7%, and 55.5%, respectively. These frequencies are in the ranges of the percentages reported for levels II, III, and IV LNM, respectively, 27–65%, 57–82%, and 41–82% in a meta‐analysis by Eskander et al. 30. In addition, most patients (137/220, 62.3%) in this study had multiple‐level metastases, which is consistent with the values reported in previous studies 16, 22. On the basis of the above reasons and the prognostic significance, it is universally agreed that therapeutic LND should include at least a comprehensive but selective LND of levels II–IV. However, whether therapeutic LND should routinely include level V lymphadenectomy remains controversial 13, 14, 15, 16, 17, 18, 19, 20, 21, 22.

In our study, level V LNM in solitary PTC with clinically LLNM was the least frequent (12.7%) type of metastasis. The rate was slightly lower than that reported in the 15 articles included in the meta‐analysis by Eskander et al. 30. This difference may be attributable to the fact that our study only included patients with solitary PTC. Moreover, our study showed that the mean number of excise and metastatic lymph nodes and the median metastatic lymph node ratio were far lower in level V than that in other levels in the lateral compartment. The risk of postoperative morbidity (especially injury to the spinal accessory nerve and cervical plexus) that can lead to increased morbidity and affect the quality of life increases with the extent of LND. Therefore, the necessity of routine level V dissection has been questioned 13, 14, 15. Furthermore, because level V LNM are comparatively rare, some researchers have suggested that routine level V dissection is not necessary for patients with PTC and lateral cervical lymphadenopathy 16, 17, 18. However, others hold the opposite view according to the high rates of metastasis seen with this cancer 19, 20, 21, 22. On the basis of the comparatively low frequency of level V LLNM in this study and the risk of postoperative complications, we propose that therapeutic LND should not routinely include level V lymphadenectomy except when level V LLNM is suspected on the basis of preoperative examination, such as US and CT, or there are associated risk factors.

Level V LNM may not be identified reliably on preoperative US and/or CT 17, 18. Therefore, we further explored the possible risk factors for level V LNM to guide level V lymphadenectomy. Our study demonstrated that the mean size of the primary tumor in patients with level V LNM was greater than that in patients without level V LNM; moreover, tumor size >10 mm was associated with level V LNM. In contrast, this association was not found by previous studies 17, 18, 19, 21, which might have been affected by multifocality. Of 27 patients with level V LNM, only one patient had a tumor size <10 mm (microcarcinoma). Thus, in solitary papillary thyroid microcarcinoma with clinically LLNM, the LNM seldom spread to level V. Zhang et al. 21 found that extrathyroidal extension was an independent risk factor for level V LNM. However, our study showed that extrathyroidal extension was a risk factor, although not an independent risk factor, for level V LNM. Our study also showed that level V LNM was associated with simultaneous level II–IV LNM, which is similar to that reported in the study by Lim et al. 16, Shim et al. 17, and Zhang et al. 21. Furthermore, Shim et al. 17 and Zhang et al. 21 found that simultaneous level II–IV LNM was also an independent risk factor for level V LNM. Our study demonstrated that level V LNM was not associated with ipsilateral CLNM, but an ipsilateral CLNM ratio ≥50%, contralateral LNM, and bilateral CLNM were risk factors for level V LNM. One important finding in our study was that contralateral CLNM was an independent risk factor for level V LNM. Therefore, we suggest that, when patients with solitary PTC and clinically LLNM present with the above mentioned associated risk factors (especially contralateral CLNM), level V lymphadenectomy may be considered in therapeutic LND. To the best of our knowledge, this is the first study to investigate the risk value of CLNM with separate compartments for level V LNM in solitary PTC with clinically LLNM.

There are some potential limitations of this study. Because this was a retrospective study based on the review of medical records, wherein clinical LLNM were not partitioned by preoperative US according to the lateral neck level, the detected value of US for each level LNM could not be evaluated. Moreover, the specimens of level V LNM were not routinely divided into levels V_a_ and V_b_ by the surgeon; therefore, levels V_a_ and V_b_ could not be assessed separately. Furthermore, other risk factors such as histological subtype, lymphovascular invasion, distant metastasis, and lymph nodal volume were not included because they were not routinely reported in the pathological report in our institution. Finally, patients who underwent elective (<4 levels) or prophylactic LND were not enrolled, which may have weakened the impact of some of the study outcomes.

## Conclusions

This study showed a high prevalence of level II–IV LNM and a relatively low prevalence of level V LNM in patients with solitary PTC and clinically LLNM. A comprehensive selective level II–IV LND should be included in the therapeutic LND for solitary PTC patients with clinically LLNM. Routine level V lymphadenectomy may not be necessary for the treatment of solitary PTC in patients with clinically LLNM unless level V LNM is suspected according to preoperative findings on examination or associated risk factors, especially contralateral CLNM, are present. However, further prospective studies will be warranted to elucidate risk factors for level V LNM and to evaluate the impact of level V lymphadenectomy on prognostic outcomes in these patients.

## Conflict of Interest

None declared.
